# Bile pigment in small-bowel water content may reflect bowel habits: a retrospective analysis of a capsule endoscopy imaging series

**DOI:** 10.1186/s12876-020-01382-0

**Published:** 2020-07-23

**Authors:** Taiki Aoyama, Akira Fukumoto, Kenjiro Shigita, Naoki Asayama, Shinichi Mukai, Shinji Nagata

**Affiliations:** 1 Department of Gastroenterology, Hiroshima City Asa Citizens Hospital, 2-1-1 Kabe-minami, Asakita-ku, Hiroshima, 731-0293 Japan; 2 Department of Endoscopy, Hiroshima City Asa Citizens Hospital, 2-1-1 Kabe-minami, Asakita-ku, Hiroshima, 731-0293 Japan

**Keywords:** Capsule endoscopy, Bile pigments, Abnormal bowel habits, bile acid, small-bowel physiology

## Abstract

**Background:**

Pigmented bile salts darken the small-bowel lumen and are present with bile acid, which is involved in the development of bowel habits. The small-bowel water content (SBWC) in the ileum could represent the colonic environment, but no studies have focused on this feature. However, measurement of crude SBWC can be challenging because of the technical difficulty of the endoscopic approach without preparation. Our aim was to evaluate optically active bile pigments in the SBWC of patients with abnormal bowel habits using capsule endoscopy (CE) to investigate the impact of bile acid on bowel habits.

**Methods:**

The study population included 37 constipated patients, 20 patients with diarrhea, and 77 patients with normal bowel habits who underwent CE between January 2015 and May 2018. Patients with secondary abnormal bowel habits were excluded. In addition to conventional imaging, we used flexible spectral imaging color enhancement (FICE) setting 1 imaging, in which the effects of bile pigments on color are suppressed. Intergroup color differences of SBWC in the ileum (ΔE) were evaluated from conventional and FICE setting 1 images. Color values were assessed using the CIE L*a*b* color space. Differences in SBWC lightness (black to white, range 0–100) were also evaluated.

**Results:**

The ΔE values from the comparison of conventional images between patients with constipation and with normal bowel habits and between patients with diarrhea and with normal bowel habits were 12.4 and 11.2, respectively. These values decreased to 4.4 and 3.3, respectively, when FICE setting 1 images were evaluated. Patients with constipation and diarrhea had significantly brighter (34.4 versus 27.6, *P* < .0001) and darker (19.6 versus 27.6, *P* < .0001) SBWC lightness, respectively, than patients with normal bowel habits. The FICE setting 1 images did not reveal significant differences in SBWC lightness between those with constipation and with normal bowel habits (44.1 versus 43.5, *P* = .83) or between those with diarrhea and with normal bowel habits (39.1 versus 43.5, *P* = .20).

**Conclusions:**

Differences in SBWC color and darkness in the ileum appear to be attributable to bile pigments. Therefore, bile pigments in SBWC may reflect bowel habits.

## Background

Functional gastrointestinal disorders (FGIDs), also referred to as disorders of gut-brain interaction in Rome IV [1], negatively affect quality of life and impose a socioeconomic burden [2]. Although several endocrine, neurologic, and microbiome factors may result in FGID [3], obvious causes are not always evident. A deficiency of bile acid (BA) can contribute to the pathophysiology of constipation [4], while increasing intracolonic concentrations of BA can accelerate spontaneous peristalsis [5], with excessive BA resulting in loose stool consistency [6]. In the gastrointestinal (GI) tract, BA plays a role in the modulation of fluid and electrolyte absorption and the regulation of GI motility [7–9]. Through these functions, BA is important for the development of bowel habits. However, we were unable to find any previous studies that have investigated the contribution of BA in determining small-bowel water content (SBWC). To evaluate the role of BA in the development of abnormal bowel habits, we investigated SBWC just proximal to the colon.

Capsule endoscopy (CE) is an established modality, which is now considered the first choice for examination of small-bowel lesions. However, up to 30% of these lesions may be missed because of inherent limitations of the technology, especially when there is increased gut motility and/or a lesion is captured in only one frame because of the rapid passage of the capsule through the bowel [10, 11]. To increase the detection of small-bowel lesions that could be missed using conventional imaging, flexible spectral imaging color enhancement (FICE) has been developed [12]. FICE decomposes conventional CE images into 3 specific wavelengths (red, green, and blue) and then directly reconstructs the image. The assessor can easily switch between viewing conventional images and the 3 different settings of FICE images. The wavelengths for FICE settings that are used in the evaluation of CE images are as follows: setting 1 (red, 595 nm; green, 540 nm; blue, 535 nm), setting 2 (red, 420 nm; green, 520 nm; blue, 530 nm), and setting 3 (red, 595 nm; green, 570 nm; blue, 415 nm) [13]. Among the 3 FICE settings, FICE setting 1 is optimized to reduce the interference of bile pigments, which contain bilirubin, which darkens the small bowel and impairs visibility. Specifically, as the wavelength for maximum absorbance of bilirubin is 455 nm, the spectrum of FICE setting 1 is designed to avoid the 400- to 500-nm wavelength range [14].

Increasing attention is being paid to the assessment of the small bowel, with the identification of small-bowel lesions being used to assess the occurrence of systemic diseases and other conditions [15–17]. The SBWC in the ileum could be a significant indication of the colonic environment, although measurement of crude SBWC can be challenging due to the technical difficulty of ileum endoscopy without preparation. It is well-known that bilirubin excretion could parallel that of BA [18, 19]. Moreover, bile pigment can be considered to be colorimetrically dominant in the SBWC [20]. Therefore, the present retrospective study aimed to evaluate optically active bile pigments in the SBWC of patients with abnormal bowel habits using CE and to indirectly assess whether BA involvement is associated with abnormal bowel habits. The method of color analysis used in the study has been assessed and validated by several endoscopists in previous studies demonstrating the usefulness of image-enhanced endoscopy for gastrointestinal neoplasms [21, 22].

## Methods

### Aim of the study

This retrospective study aimed to analyze the contribution of BA in the SBWC in the ileum to the development of abnormal bowel habits using optical effects observed in color analysis of capsule endoscopy images.

### Study population

A total of 223 consecutive patients who underwent small-bowel exploration by CE between January 1, 2015, and May 30, 2018, were included. After excluding 15 patients who presented with blood or opacity in the small bowel, 17 were excluded on the basis of medication use (sodium chenodeoxycholate in 7 and magnesium oxide in 10, both of which can influence SBWC). Following these exclusions, 172 patients were eligible for image evaluation. Patients with abnormal bowel habits secondary to other conditions were then excluded. After 29 patients with enteritis or colitis including inflammatory bowel disease were excluded, 5 patients with specific systemic diseases were also excluded. These diseases were Parkinson disease in 1 patient, thyroid function abnormalities in 3 patients, and diabetic neuropathy in 1 patient. An additional 2 patients who underwent bowel resection and 2 who underwent cholecystectomy were excluded. No patients with advanced colorectal cancer were included in the initial population.

**Fig. 1:**
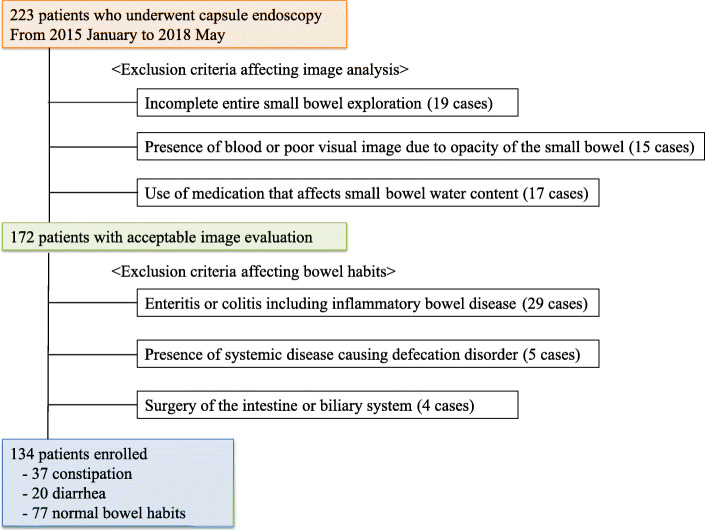
Patient enrollment flowchart. Medications considered to affect small-bowel water content were sodium chenodeoxycholate, magnesium oxide, bile acid sequestrants, elobixibat, lubiprostone, and linaclotide. Medications considered to affect small-bowel motility were opioids and dopamine antagonists. Systemic diseases that cause bowel-habit abnormalities leading to study exclusion were Parkinson disease, thyroid function abnormalities, chronic pancreatitis, and diabetic neuropathy

The study population included 134 patients (male, 74; female, 60; mean age, 64.6 years; range, 10–88 years) who underwent CE at Hiroshima City Asa Citizens Hospital, between January 2015 and May 2018. Of these 134 patients, 37 were constipated, 20 had diarrhea, and 77 normal bowel habits (not being categorized into either the constipation or diarrhea group). Patients were classified on the basis of their predominant bowel habits, which were assessed by a questionnaire administered before CE examination. The initial indications for CE were suspected bleeding from the small bowel, unexplained abdominal symptoms, abnormal findings on other abdominal imaging techniques, and abnormal laboratory findings. The flowchart of patient enrollment is shown in Fig. 1.

### Capsule endoscopy

For examination of the small bowel, CE was performed using a PillCam™ SB3 video capsule (Medtronic, Dublin, Ireland). The capsules were swallowed with a solution of dimethicone after a 12-h overnight fast, without any other preparation. Once the capsules were swallowed, patients continued with their routine daily activities. Patients were able to drink clear liquids and eat a light meal 2 and 4 h after swallowing the capsules, respectively. After 9 h, the sensor arrays and recording devices were removed, and images were analyzed using Rapid™ for Pillcam™ software that was installed on a Rapid Workstation R8.0 (Medtronic), with FICE software incorporated. Captured CE images were reviewed and analyzed, independent of clinical background, by 2 endoscopists who had previously reviewed CE images of more than 500 cases. Evaluation of the images was based on mutual agreement. The entire small bowel was divided into halves on the basis of the capsule transit time, with images in the first part considered to be of the jejunum and those from the latter part from the ileum.

### Image acquisition

The latter half of the small bowel, defined as the ileum in the present study, was further divided into five equal segments on the basis of the capsule transit time. Five eligible CE images showing small-bowel water pooled in the lumen were selected from each segment of the ileum from each patient. These images were extracted from the regions whose colors were dominant in the color bar on the Rapid Workstation. Images with optical artifacts and/or significant amounts of floating residue were not considered in the selection. The FICE setting 1 images were acquired by converting the selected conventional CE images. Five pairs of conventional and corresponding FICE setting 1 images were selected from the ileum of each patient.

### Image processing

**Fig. 2:**
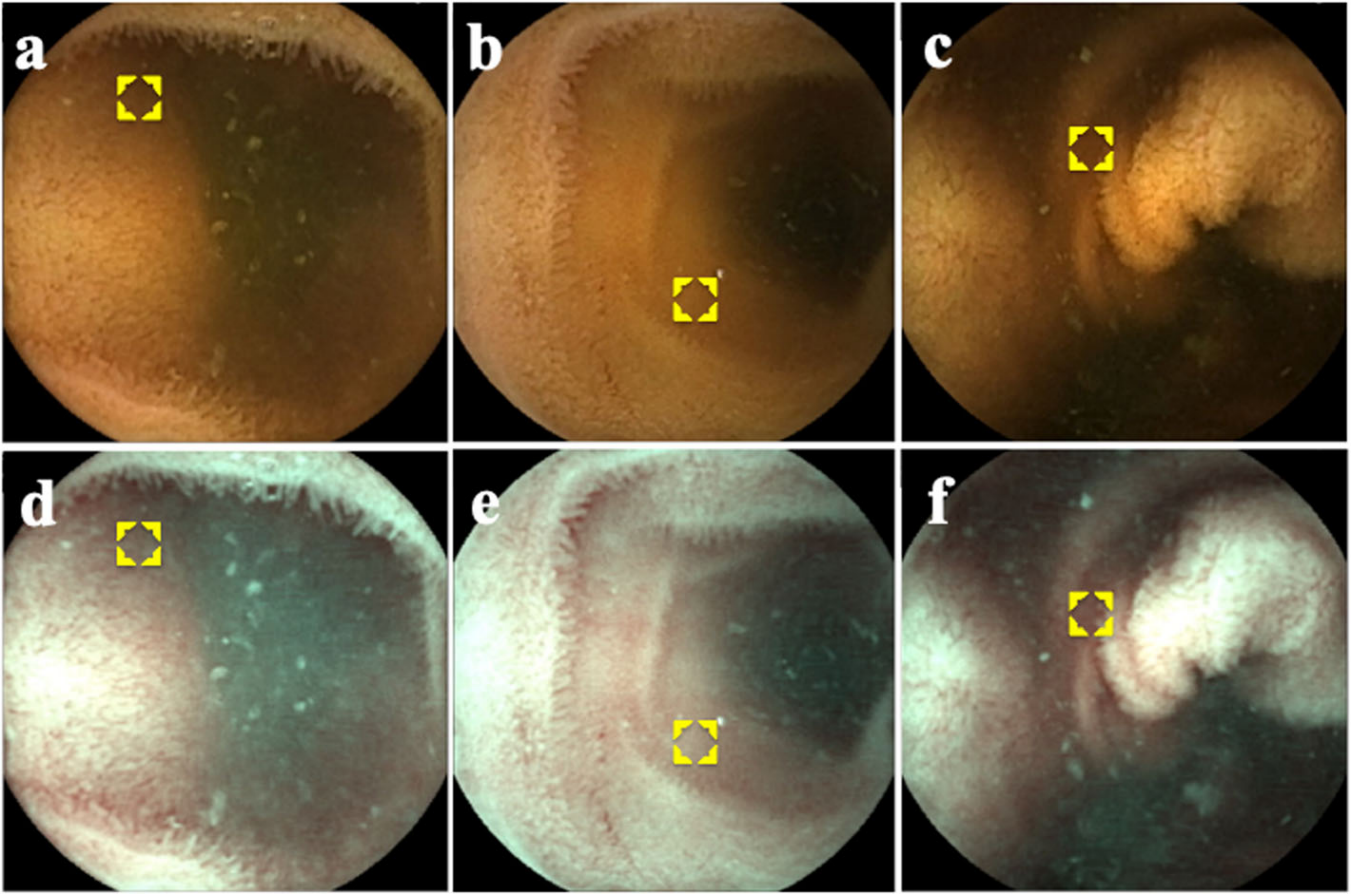
Small-bowel water content in the ileum. Representative raw conventional images from (**a**) a patient with normal bowel habit, (**b**) a patient with constipation, and (**c**) a patient with diarrhea. The corresponding flexible spectral imaging color enhancement setting 1 images are shown in (**d**), (**e**), and (**f**), respectively. Yellow boxes indicate the regions of interest

Color processing and image analysis were performed using Adobe Photoshop Elements 10 (Adobe Systems, San Jose, CA, USA). The region of interest (ROI) was set at the area of pooled water with 1- to 2-cm thickness between the capsule and the facing mucosa in the lumen to avoid halation and shadowing in order to represent the entire SBWC in the image (yellow boxes in Fig. 2). The position of the ROI was set on the conventional image after agreement between the 2 endoscopists and applied to the corresponding FICE setting 1 image. Color information (red, blue, and green) was extracted from the ROI, then converted to the Commission Internationale d’Eclairage (CIE) L*a*b* color values [23]. Five pairs of conventional images and corresponding FICE setting 1 images were processed in this manner, and the average L*a*b* values were calculated for each pair. These were considered representative values for each patient and were calculated for every patient in the study (Additional file 1).

### Color analysis

**Fig. 3:**
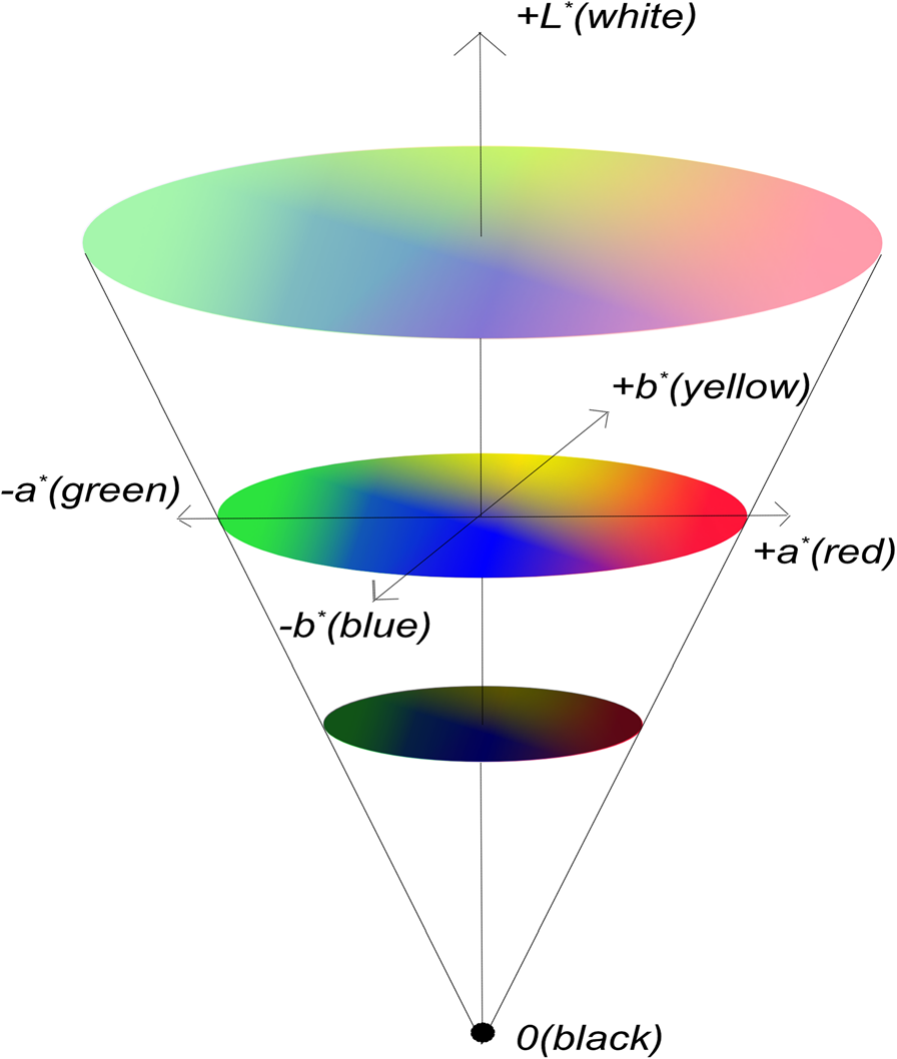
Commission Internationale d’Eclairage (CIE) L*a*b* color. Color is expressed as 3 numerical values, L* for lightness and a* and b* for the green-red and blue-yellow color components, respectively. The system was designed to be perceptually uniform with respect to human color vision. The space itself is a 3-dimensional real-number space; therefore, any color variation can be expressed in L*a*b* coordinates

The color value for each image was expressed using the 3-dimensional color parameters L* (black to white; range, 0–100), a* (green to red; range, 128–127), and b* (blue to yellow; range, 128–127) (Fig. 3). The relative perceptual difference between any 2 colors can be approximated by the color distance between them. Color differences (ΔE) of SBWC between groups were calculated using the following equation [24]:

$$ \Delta \mathrm{E}=\left[\left(\Delta {\mathrm{L}}^{\ast}\right)2+\left(\Delta {\mathrm{a}}^{\ast}\right)2+\left(\Delta {\mathrm{b}}^{\ast}\right)2\right]1/2. $$

The ΔE value was calculated for each comparison and classified into units of color difference according to the evaluation criteria of the National Bureau of Standards (NBS), using the following equation [21, 25]:

$$ \mathrm{NBS}\ \mathrm{units}=\Delta \mathrm{E}\times 0.92. $$

The NBS defines the units of color difference as follows: 0–0.5 = trace; 0.5–1.5 = slight; 1.5–3.0 = noticeable; 3.0–6.0 = appreciable; 6.0–12.0: much; ≥12.0 = very much.

During image selection, acquisition, processing, and color analysis, the endoscopists were blinded to patients’ clinical information.

### Study comparisons

Patients were classified into 3 groups according to their answers to the questionnaire administered before CE examination: constipation, diarrhea, and normal bowel habits. The questionnaire asked details regarding stool frequency and bowel habits over the last 3 months, based on information maintained by each patient in a daily diary. Patients were provided with the definition of constipation and diarrhea before completing the questionnaire to improve the reliability of the information provided. Constipation was defined as fewer than 3 spontaneous bowel movements per week or difficulty in > 25% of defecation events. Diarrhea was defined as loose or watery stools occurring in > 25% of defecation events [26]. The questionnaire also asked about the presence of abdominal pain related to defecation. “Presence” of pain was defined as having pain at least 1 day per week for the last 3 months.

The patients’ demographic and clinical characteristics were evaluated. These included age, sex, alcohol consumption, and smoking habits; comorbidities including hypertension, diabetes mellitus, hyperlipidemia, cardiovascular disease, chronic liver disease, and chronic kidney disease; and current medications, including nonsteroidal anti-inflammatory drugs, antithrombotic agents, and proton pump inhibitors. The mean defecation frequency of each group was calculated, and the capsule transit time in the stomach and small bowel was evaluated.

The ΔE values for the SBWC of the ileum of the groups with constipation and with diarrhea were compared to those of the group with normal bowel habits. The median values of L*a*b* and the NBS units of ΔE were compared between groups for both the conventional and FICE setting 1 images.

The average SBWC lightness (L*) of the ileum for the groups with constipation and with diarrhea were also compared to that of the group with normal bowel habits for both conventional and FICE setting 1 images.

### Statistical analyses

Student’s *t* test was used to compare quantitative data using JMP version 9 (SAS Institute, Cary, NC, USA). The chi-squared test, with a 3 × 2 contingency table, was used to compare categorical data using Microsoft Excel 2008 for Mac (Microsoft, Redmond, WA, USA), with Yates’ correction when required. All of the tests were 2-sided, and *P* < .05 was considered statistically significant.

## Results

The clinical characteristics of all groups are presented in Table 1. Univariate analysis indicated no statistically significant differences in demographics, psychosocial histories, comorbidities, or medications among the groups. The mean frequency of defecation was 1.1 times daily for the group with normal bowel habits, 0.5 times daily for the group with constipation, and 3.0 times daily for the group with diarrhea. Abdominal pain was present in 11 of patients in the group with normal bowel habits, 6 in the group with constipation, and 4 in the group with diarrhea. The mean [standard deviation; SD] capsule transit times in the stomach were not significantly different either between the groups with constipation and with normal bowel habits (50.0 [51.2] versus 33.6 [43.6], *P* = .08) or between the groups with diarrhea and with normal bowel habits (36.1 [40.3] versus 33.6 [43.6], *P* = .83). Same as the stomach, the mean capsule transit time in the small bowel were not significantly different either between the groups with constipation and with normal bowel habits (307.0 [117.9] versus 288.1 [109.7], *P* = .50) or between the groups with diarrhea and normal bowel habits (306.9 [94.3] versus 288.1 [109.7], *P* = .37).

**Table 1:** Baseline demographic and clinical patient characteristics

	Defecation phenotype			***P*** value^**d**^
	Normal bowel habits^**a**^***n*** = 77	Constipation^**a**^***n*** = 37	Diarrhea^**a**^***n*** = 20	
**Demographics**				
Age ≥ 75 years	28 (10)	10 (27)	5 (25)	.64
Male sex	44 (57)	19 (51)	11 (55)	.91
**Psychosocial history**				
Smoking	11 (14)	8 (22)	4 (20)	.77
Alcohol consumption	27 (35)	11 (30)	9 (45)	.68
**Comorbidity**				
Hypertension	37 (48)	20 (54)	6 (30)	.34
Diabetes mellitus	8 (10)	6 (16)	5 (25)	.41
Hyperlipidemia	19 (25)	5 (14)	4 (20)	.52
Cardiovascular disease	17 (22)	12 (32)	3 (15)	.46
Chronic liver disease^b^	4 (5)	1 (3)	2 (10)	.83
Chronic kidney disease^c^	26 (34)	10 (27)	4 (20)	.62
**Current medications**				
NSAIDs	7 (9)	3 (8)	2 (10)	.96
Antithrombotic agents	16 (21)	8 (22)	3 (15)	.96
PPI	20 (26)	13 (35)	7 (35)	.70

*NSAIDs* Non-steroidal anti-inflammatory drugs, *PPI* proton pump inhibitor

^a^Data are expressed as numbers and (%) of patients. ^b^Viral etiologies included infection with the hepatitis B or C virus. Non-viral etiologies included alcoholic hepatitis and autoimmune hepatitis. There were no patients with decompensated cirrhosis in the initial population. ^c^Sustained renal malfunction defined as an estimated glomerular filtration rate of < 60 mL/min.1.73 m^2^. ^d^The chi-square test for the 3 × 2 contingency table was used to compare categorical data

The ΔE values of the SBWC of the ileum for all groups are presented in Table 2. Examination of the conventional images revealed that the ΔE value between the groups with constipation and with normal bowel habits was 12.4, defined as “much” difference by the NBS rating system. Meanwhile, examination of the FICE setting 1 images, all of which were adjusted to reduce bile pigment effects, revealed that the ΔE value decreased to 4.4, defines as an “appreciable” difference. Comparison of the groups with diarrhea and normal bowel habits showed a similar trend, with the ΔE value of the conventional images being 11.2 (“much” difference) and the FICE setting 1 images suggesting ΔE to be 3.3 (“noticeable” difference).

**Table 2:** Color differences between the small-bowel water content of constipation and diarrhea patients and normal-bowel-habit patients

	Defecation phenotype	
	Constipation	Diarrhea
**Conventional images**		
ΔE (color difference) compared with normal-bowel-habit	12.4	11.2
NBS unit	11.4	10.3
Rating by NBS evaluation criteria^a^	Much	Much
**FICE setting 1 images**		
ΔE (color difference) compared with normal-bowel-habit	4.4	3.3
NBS unit	4.0	3.0
Rating by NBS evaluation criteria^a^	Appreciable	Noticeable

*Abbreviations: FICE* flexible spectral imaging color enhancement, *NBS* National Bureau of Standards

^a^ NBS classification: 0–0.5, trace; 0.5–1.5, slight; 1.5–3.0, noticeable; 3.0–6.0, appreciable; 6.0–12.0, much; 12.0 and above, very much

**Fig. 4:**
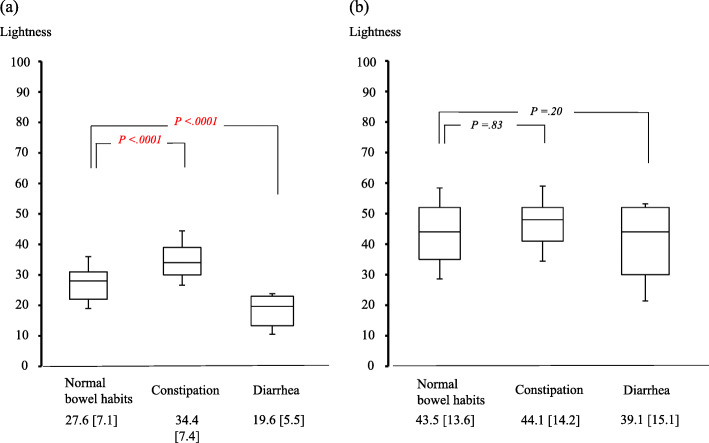
Between-group comparison of lightness of small-bowel water contents in the ileum. The scores are expressed as the mean [standard deviation]. Plots show comparisons of (**a**) conventional images and (**b**) flexible spectral imaging color enhancement setting 1 images

Comparison of the mean SBWC lightness (L*) in the ileum between groups is illustrated in Fig. 4. Examination of the conventional images indicated that the mean [SD] SBWC lightness of the group with constipation was significantly higher (appearing brighter) than that of group with normal bowel habits (34.4 [7.4] versus 27.6 [7.1], *P* < .0001). Meanwhile, the mean [SD] SBWC lightness of the group with diarrhea was significantly lower (i.e., appeared darker) than that of the group with normal bowel habits (19.6 [5.5] versus 27.6 [7.1], *P* < .0001). Examination of the FICE setting 1 images did not reveal a significant difference between the mean [SD] SBWC lightness of the groups with constipation and with normal bowel habits (44.1 [14.2] versus 43.5 [13.6], *P* = .83). Similarly, the mean [SD] SBWC lightness was not significantly different between the groups with diarrhea and with normal bowel habits (39.1 [15.1] versus 43.5 [13.6], *P* = .20).

## Discussion

The pathophysiology of FGID, including irritable bowel syndrome (IBS), is multifactorial in nature and may include altered sensation, psychosocial factors, colonic motility, and mucosal factors [1]. Interaction of these factors generates functional gastrointestinal symptoms due to “leaky” intestinal barrier, abnormal permeability, and signaling amplification [27]. In particular, luminal factors, such as BA, may induce changes in the mucosal, motor, and sensory functions [28]. Interaction of the microbiota with dietary constituents that are mixed with BA can generate biologically active molecules that influence gut motility and secretion and could play a fundamental role in the formation of bowel habits [3].

In the present study, we aimed to indirectly evaluate the contribution of BA to abnormal bowel habits in the ileum, which, to the best of our knowledge, has not been the focus of any published studies to date. To this end, we used an optical approach involving CE that did not require sampling of the small-bowel water and subsequent biochemical analyses. We evaluated the BA involvement of SBWC indirectly by assessing the darkening of the lumen, which can be attributed to bile pigments. This was achieved by analyzing the color difference between conventional and FICE setting 1 images and performing between-group comparisons. The use of all 3 FICE settings has not been reported to significantly improve the overall delineation or detection rate of small-bowel lesions; however, the usefulness of FICE setting 1 has been demonstrated in the detection of vascular lesions [29]. Moreover, the lesion-detection rate using FICE setting 1 has been reported to be unaffected by the presence of bile pigments [30]. This suggests that the use of this setting can suppress the effects of bile pigment on the color of SBWC and enhance the appearance of reddened lesions. For these reasons, the use of FICE setting 1 was deemed clinically appropriate for the present study.

Quantitative analysis of the color difference was carried out by assessing the CIE L*a*b* color values—an approach that has previously been used to demarcate lesions from the surrounding mucosa during endoscopy [21, 31]. In the present study, the color differences (ΔE) of the SBWC in the ileum between the groups with constipation and with normal bowel habits were found to be 12.4 and 4.4, respectively, using conventional and FICE setting 1 images. Similarly, the ΔE values between the groups with diarrhea and with normal bowel habits were 11.2 and 3.3, respectively, calculated from conventional and FICE setting 1 images. The ΔE values decreased for both comparisons when the FICE setting 1 (minimizing bile-pigment interference) images were used; therefore, these differences were deemed to be produced by bile pigments in the SBWC. Significant ΔE values were observed when significant differences in SBWC lightness were observed. In other words; the SBWC of constipated patients appeared brighter than that of patients with normal bowel habits, implying lower concentrations of bile pigments associated with constipation. Similarly, the SBWC of diarrhea patients appeared darker, implying higher concentrations of bile pigments than that found in patients with normal bowel habits.

Our study included 11 patients with abdominal pain in the group with normal bowel habits, 6 in the group with constipation, and 4 in the groups with diarrhea. These patients could be categorized as having IBS according to Rome IV. However, in the clinic, it may not be possible to separate the disorders exactly into separate entities, for example, differentiating IBS-C (constipation) from functional constipation and IBS-D (diarrhea) from functional diarrhea. Thus, Rome IV considers that these disorders exist on a continuum rather than in isolation [32, 33].

Our results are supported by the study of Shin et al. [34], who compared the traits of IBS with the fecal BA levels of IBS patients and healthy volunteers. They reported that the fecal levels of BA were significantly higher in subjects with IBS-D. Moreover, they found that subjects with IBS-C had lower fecal levels of BA than healthy volunteers. Normalization of bowel function, either by BA supplementation or sequestration in IBS-C and IBS-D, respectively, confirms that characterization of fecal BA composition may be necessary to clarify the pathogenic role of BA [6, 35]. In IBS-D or functional diarrhea, increased fecal BA may result from increased synthesis of BA, possibly due to deficient negative-feedback inhibition of BA synthesis or genetic variation [36, 37]. These facts suggest that BA involvement in the small bowel still has an impact on the development of bowel habit, although 95% of BA is recycled in the terminal ileum. Elobixibat is a minimally absorbed ileal bile acid–transport inhibitor and is used clinically to treat constipation by augmenting the level of BA that flows to the colon. Increased dosage causes an increased frequency of diarrhea and corresponding abdominal symptoms [38], as BA can affect stool consistency or liquefy the stool.

Although FGID may be associated with motility abnormalities, such as rapid intestinal transit in IBS-D [39], FGID is defined using a symptom-based, rather than a motility-based, approach [1]. The capsule transit time in the small bowel showed no significant differences among groups in the present study, and thus, it did not seem to be correlated with bowel habits.

Our study has some limitations. First, we did not compare the SBWC among the patients in vitro. An association between the indirect assessment of BA concentration and the real BA concentration in the ileum is lacking in the present study. Second, we did not evaluate the Bristol Stool Scale [40]. The reason for this was because we did not aim to subclassify IBS patients but rather to simply compare the study subjects with abnormal bowel habits. Third, this was a retrospective, single-center study. We recognize that local biological factors, including genetics, microbiome, environmental hygiene, cytokines, and central nervous system effects, could have an impact on symptom generation, manifestation, and interpretation. Therefore, we acknowledge the shortage of study population to produce enough evidence, and a large-scale prospective study is needed to address these limitations.

## Conclusions

Pigmentation of the SBWC in the ileum could be formed by bile. Moreover, the degree of bile pigmentation may affect bowel habit.

## Supplementary information

**Additional file 1.** Median of representative L^*^a^*^b^*^ values of the small-bowel water content in the groups with constipation, with diarrhea, and with normal bowel habits.

**Additional file 2.** The effect of flexible spectral imaging color enhancement setting 1 on the influence of bile pigments. (a) Original bile juice, (b) white light image, (c) flexible spectral imaging color enhancement setting 1 image, (d) two-fold dilution bile juice, (e) white light image, and (f) flexible spectral imaging color enhancement setting 1 image.

## Data Availability

The datasets used and/or analyzed during the current study are available from the corresponding author on reasonable request.

## Abbreviations

BA: bile acid
SBWC: small-bowel water content
CE: capsule endoscopy
FICE: flexible spectral imaging color enhancement
FGIDs: functional gastrointestinal disorders
GI: gastrointestinal
IRB: institutional review board
ROI: region of interest
CIE: Commission Internationale d’Eclairage
NBS: National Bureau of Standards
SD: standard deviation
IBS: irritable bowel syndrome
PPI: proton pump inhibitor
