# Supplementary material for: Bile pigment in small-bowel water content may reflect bowel habits: a retrospective analysis of a capsule endoscopy imaging series
Source: BMC Gastroenterol. 2020 Jul 23;20:237. doi: 10.1186/s12876-020-01382-0 (PMC7376737; doi:10.1186/s12876-020-01382-0)
Supplement: Supplementary file 1 — Additional file 1. Median of representative L*a*b* values of the small-bowel water content in the groups with constipation, with diarrhea, and with normal bowel habits. [file 12876_2020_1382_MOESM1_ESM.docx]

**Additional file 1. Median of representative L^*^a^*^b^*^ values of the small-bowel water content in the group with constipation, with diarrhea, and with normal bowel habits**

|  | **Defecation phenotype** | | |
| --- | --- | --- | --- |
|  | **Normal bowel habits** | **Constipation** | **Diarrhea** |
| **Conventional images** | (29, 8, 26) | (35, 17, 32) | (21, 3, 20) |
| **FICE setting 1 images** | (46, 1, 11) | (49, 4, 10) | (47, -2, 10) |

Data was expressed as (L^*^a^*^b^*^).
