# Supplementary figures and images for: Bile pigment in small-bowel water content may reflect bowel habits: a retrospective analysis of a capsule endoscopy imaging series
Source: BMC Gastroenterol. 2020 Jul 23;20:237. doi: 10.1186/s12876-020-01382-0 (PMC7376737; doi:10.1186/s12876-020-01382-0)

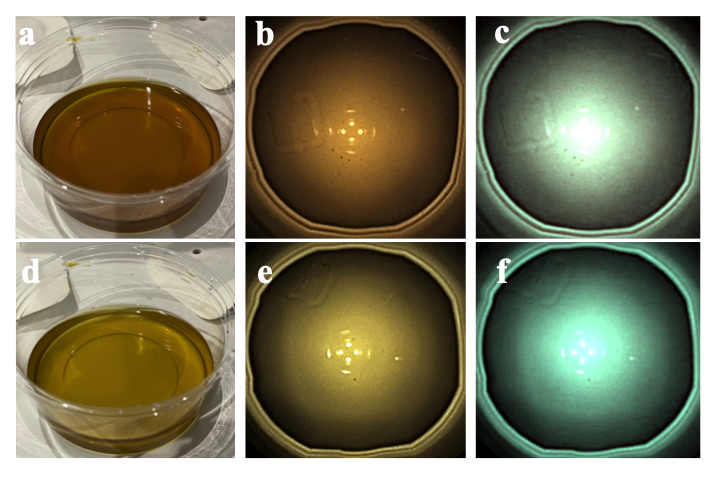

Supplement: Supplementary file 2 — Additional file 2. The effect of flexible spectral imaging color enhancement setting 1 on the influence of bile pigments. (a) Original bile juice, (b) white light image, (c) flexible spectral imaging color enhancement setting 1 image, (d) two-fold dilution bile juice, (e) white light image, and (f) flexible spectral imaging color enhancement setting 1 image. [file 12876_2020_1382_MOESM2_ESM.tiff]
